# RNA language model and graph attention network for RNA and small molecule binding sites prediction

**DOI:** 10.1093/bioinformatics/btaf447

**Published:** 2025-08-06

**Authors:** Saisai Sun, Jianyi Yang, Lin Gao, Pengyong Li, Yumeng Liu

**Affiliations:** School of Computer Science and Technology, Xidian University, Xi’an, Shaanxi 710126, China; MOE Frontiers Science Center for Nonlinear Expectations, Research Center for Mathematics and Interdisciplinary Sciences, Shandong University, Qingdao, Shandong 266237, China; School of Computer Science and Technology, Xidian University, Xi’an, Shaanxi 710126, China; School of Computer Science and Technology, Xidian University, Xi’an, Shaanxi 710126, China; College of Big Data and Internet, Shenzhen Technology University, Shenzhen, Guangdong 518118, China

## Abstract

**Motivation:**

The structural complexities enable RNA to serve as a versatile molecular scaffold capable of binding small molecules with high specificity. Understanding these interactions is essential for elucidating RNA’s role in disease mechanisms and developing RNA-targeted therapeutics. However, predicting RNA-small molecule binding sites remains a significant challenge due to their conformational flexibility, structural diversity, and the limited availability of high-resolution structural data.

**Results:**

In this study, we propose RLsite, a novel computational framework integrating pre-trained RNA language models with graph attention networks (GAT) to predict small-molecule binding sites on RNA. Our method effectively captures both sequential and structural features of RNA by leveraging large-scale RNA sequence data to learn intrinsic patterns and processing graph-based RNA structures to highlight key topological and spatial features. Compared to existing methods, RLsite demonstrates superior accuracy, generalizability, and biological relevance, achieving a Precision of 0.749, a Recall of 0.654, an MCC of 0.474, and an AUC of 0.828 on the public test set, which significantly outperforms the previous models, such as CapBind (an AUC of 0.770), MultiModRLBP (an AUC of 0.780), and RNABind (an AUC of 0.471). Notably, a case study of the PreQ1 riboswitch has achieved strong predictive performance (AUC = 0.97, Recall = 0.9), and its predicted binding sites have been confirmed experimentally. These results underscore our method as a potentially powerful tool for RNA-targeted drug discovery and advancing our understanding of RNA-ligand interactions.

**Availability and implementation:**

The resource codes and data can be accessed at https://github.com/SaisaiSun/RLsite.

## 1 Introduction

Ribonucleic acid (RNA) plays a fundamental role in various cellular processes, extending beyond its traditional function as a genetic messenger. It actively participates in gene expression regulation, protein synthesis, and cellular defense mechanisms against foreign nucleic acids (Yu *et al.* 2020). Due to its structural diversity and functional complexity, RNA serves as a key regulator of numerous biological pathways. In recent years, dysregulation of RNA expression and function has been closely associated with the development and progression of various diseases, including cancer, viral infections, cardiovascular disorders, and neurodegenerative conditions (Shao and Zhang 2020, Coan *et al.* 2024, Nemeth *et al.* 2024). Given its critical involvement in disease mechanisms, RNA has emerged as a promising target for drug discovery and therapeutic interventions. RNA-targeted therapeutics, such as small-molecule compounds, antisense oligonucleotides, and RNA interference (RNAi) technologies, offer potential strategies to modulate RNA function, affecting processes such as splicing, stability, translation, and overall gene regulation to mitigate disease progression (Yu *et al.* 2020, Nemeth *et al.* 2024).

Accurately predicting RNA-small molecule binding sites is crucial for uncovering the biological roles of RNA and advancing the development of RNA-targeted therapeutics (Chen *et al.* 2024, Jung *et al.* 2025). Understanding how specific RNA molecules interact with small molecules provides valuable insights into disease mechanisms, enabling the design of targeted small-molecule drugs that precisely modulate RNA function (Ghidini *et al.* 2021, Wang *et al.* 2023a,b; Wei *et al.* 2023, Jung *et al.* 2025). Additionally, elucidating these interactions facilitates the identification of novel drug targets, paving the way for more effective therapeutic strategies. As a result, research on RNA-small molecule interactions plays a vital role in drug discovery. Traditional experimental approaches for identifying these interactions are often labor-intensive, costly, and time-consuming, whereas computational prediction methods provide a more efficient and cost-effective alternative.

Despite its significant potential, studying RNA-small molecule interactions presents considerable challenges (Childs-Disney *et al.* 2022, Zeng *et al.* 2022, He *et al.* 2023, Zhuo *et al.* 2025). The structural complexity and dynamic nature of RNA molecules make it difficult to accurately identify their binding sites with small molecules (Rizvi *et al.* 2020). Moreover, current methods often fail to meet the demands of large-scale RNA-small molecule interaction analyses (Liu 2019). Therefore, developing novel computational and experimental approaches is essential to deepen our understanding of RNA function and accelerate the discovery of innovative RNA-based therapeutics (Ganser *et al.* 2018, Chen *et al.* 2024, Zhuo *et al.* 2025).

Recent advancements in computational biology, particularly deep learning, have significantly improved RNA-small molecule binding site prediction techniques. Various computational approaches have been developed for this purpose, including Rsite (Zeng *et al.* 2015), Rsite2 (Zeng and Cui 2016), RBind (Wang *et al.* 2018), RNAsite (Su *et al.* 2021), RLBind (Wang *et al.* 2023), RNet (Liu *et al.* 2024), CapBind (Xi *et al.* 2024), SHAMAN (Panei *et al.* 2024), MultiModRLBP (Wang *et al.* 2024a), and RNABind (Zhu *et al.* 2025). Rsite, a tertiary structure-based method, predicts functional sites by analyzing Euclidean distances between nucleotides. Rsite2 refines this approach by incorporating Hamming distance analysis in RNA secondary structures, offering a novel perspective on binding site identification. RBind uses a structural computational network that uses closeness and degree metrics to detect RNA-ligand binding sites. RNAsite introduced machine learning into this field, utilizing a Random Forest model trained on RNA sequences and structural features. RLBind further advanced RNA-ligand binding site prediction by replacing the Random Forest model with deep learning techniques. RNet provides an interpretable network-based framework for extracting precise binding site information and understanding RNA-ligand interaction dynamics. CapBind applies a multi-level capsule network to capture critical contextual information for predicting RNA-small molecule interactions. SHAMAN efficiently detects and ranks probable binding pockets based on probe occupancy and binding free energy by using small molecular probes. MultiModRLBP integrates multi-modal features using deep learning algorithms for RNA-small molecule binding site prediction. The latest method, RNABind, integrates RNA large language models (LLMs) with advanced geometric deep learning networks, enabling the encoding of both RNA sequence and structural information.

Despite the development of these methods, predicting RNA-small molecule binding sites remains a significant challenge. Most methods focus on static RNA structures and neglect the dynamic conformational changes of RNA molecules, which are crucial for accurately modeling RNA-ligand interactions (Childs-Disney *et al.* 2022). Traditional machine learning methods, such as RNAsite, utilize models like Random Forest, which are effective in feature-based classification but lack the depth required to capture the intricate dependencies within RNA-ligand interactions. While deep learning models offer improved predictive capabilities, their performance is often hindered by the limited availability of high-quality training data, restricting their ability to generalize across diverse RNA-ligand complexes. Furthermore, many computational approaches do not incorporate experimental validation, raising concerns about their reliability and applicability in real-world biological research. Addressing these limitations requires the development of models that integrate RNA structural flexibility, leverage larger and more diverse datasets, and incorporate experimental validation to enhance predictive accuracy and practical relevance.

While RNA has emerged as a promising target for therapeutic intervention, accurately predicting small-molecule binding sites on RNA remains a major computational challenge. This difficulty stems from RNA’s dynamic structural conformations and the limited availability of high-resolution RNA-ligand complex data. Existing models often overlook the interplay between sequence and tertiary structure or rely heavily on static representations, leading to suboptimal predictive performance, particularly in cases involving structurally diverse RNAs.

To address these limitations, we propose RLsite, a novel hybrid framework that integrates graph attention networks (GATs) and pretrained RNA language models to predict small-molecule binding sites. Our motivation is to leverage the structural information encoded in RNA 3D conformations alongside the contextual richness of RNA sequences learned from large-scale pretraining, thereby improving binding site prediction in both accuracy and generalizability. Our key contributions are as follows:

- A novel hybrid architecture combining a GAT for RNA structural modeling, extracting and propagating node information within RNA structures, with a pretrained RNA language model compensating for the scarcity of structural data while leveraging large-scale RNA sequence data to uncover intrinsic patterns in RNA molecules, which enabling a more comprehensive exploration of RNA-small molecule interactions. Here, the GAT operates on a static graph representation of RNA tertiary structure. It captures the local and global topological features of the RNA by allowing the model to focus on important nodes (nucleotides) and their relationships based on structural features like secondary structure, accessible surface area, and torsional angles.
- Multi-modal node representations incorporating secondary structure, surface accessibility, and torsional information to capture the biological and physicochemical properties of RNA.
- An effective feature fusion mechanism using a multilayer perceptron (MLP) to align and integrate structural and sequential embeddings into a unified latent space for classification.
- Comprehensive benchmarking across datasets and a real-world riboswitch case study, demonstrating superior performance over existing methods in RNA-small molecule binding site prediction.

To evaluate the generalization capability of RLsite, we used three independent test sets. The results indicate that RLsite outperforms competing models, particularly regarding Recall and the Matthews Correlation Coefficient (MCC). Our findings suggest that combining the large-scale pre-trained language model with GAT can significantly enhance the accuracy of RNA-small molecule binding site predictions. Furthermore, our experimental validation demonstrated that RLsite can identify drug binding sites accurately, potentially providing valuable insights into gene expression regulation and novel RNA-targeted therapeutic strategies for diseases such as neoplastic transformation.

## 2 Materials and methods

### 2.1 Benchmark datasets

Our study utilized the same training dataset as MultiModRLBP (Wang *et al.* 2024a), which integrates data from the RNAglib dataset (Mallet *et al.* 2022) and the RNAsite training set (Su *et al.* 2021). The RNAglib dataset contains 3739 RNA entries sourced from the Protein Data Bank (PDB) database (Burley *et al.* 2025), from which researchers filtered 810 RNAs with small-molecule binding information, corresponding to 1248 RNA chains. After that, RNA chains exceeding 440 nucleotides in length and containing fewer than four binding sites were excluded. This refinement resulted in a curated set of 653 RNA chains and 228 ligands, yielding a total of 1012 RNA–ligand pairs. The RNAsite training set, on the other hand, was derived from 78 RNA chains clustered into 57 groups based on structural similarity. From these, 42 clusters were selected, yielding 127 RNA-small molecule pairs. By merging the RNAglib dataset with the RNAsite training set and eliminating duplicate RNA chains, a final set of 666 RNA chains was curated. To ensure independence from the test data, chains overlapping with the public test set (T18) were removed, resulting in a training set of 561 RNA chains. To improve the model performance, we conducted a 5-fold cross-validation on our training set to identify the optimal model parameters, which are presented in Table S1, available as supplementary data at *Bioinformatics* online.

For evaluation, we utilized two publicly available test sets, T18 and T3, along with our newly constructed test set, T10. The T18 dataset, originally part of RNAsite, comprises 18 RNA chains and 21 small molecules, creating 26 RNA-small molecule pairs. The second test set, T3, was derived from RLBind’s test set but reduced by eliminating 6 RNA molecules with over 30% sequence similarity with the training set. This led to a final dataset containing 3 RNA chains and 4 ligands, forming 6 RNA-small molecule pairs. Regarding T10, it was sourced from RNAs in the PDB, specifically entries released after 2021. Following internal sequence clustering and filtering out redundant RNAs from the training set, 10 RNA chains were retained as T10. These benchmark datasets facilitated a comprehensive assessment of our model’s predictive performance.

### 2.2 Model architecture overview

**Figure 1.**
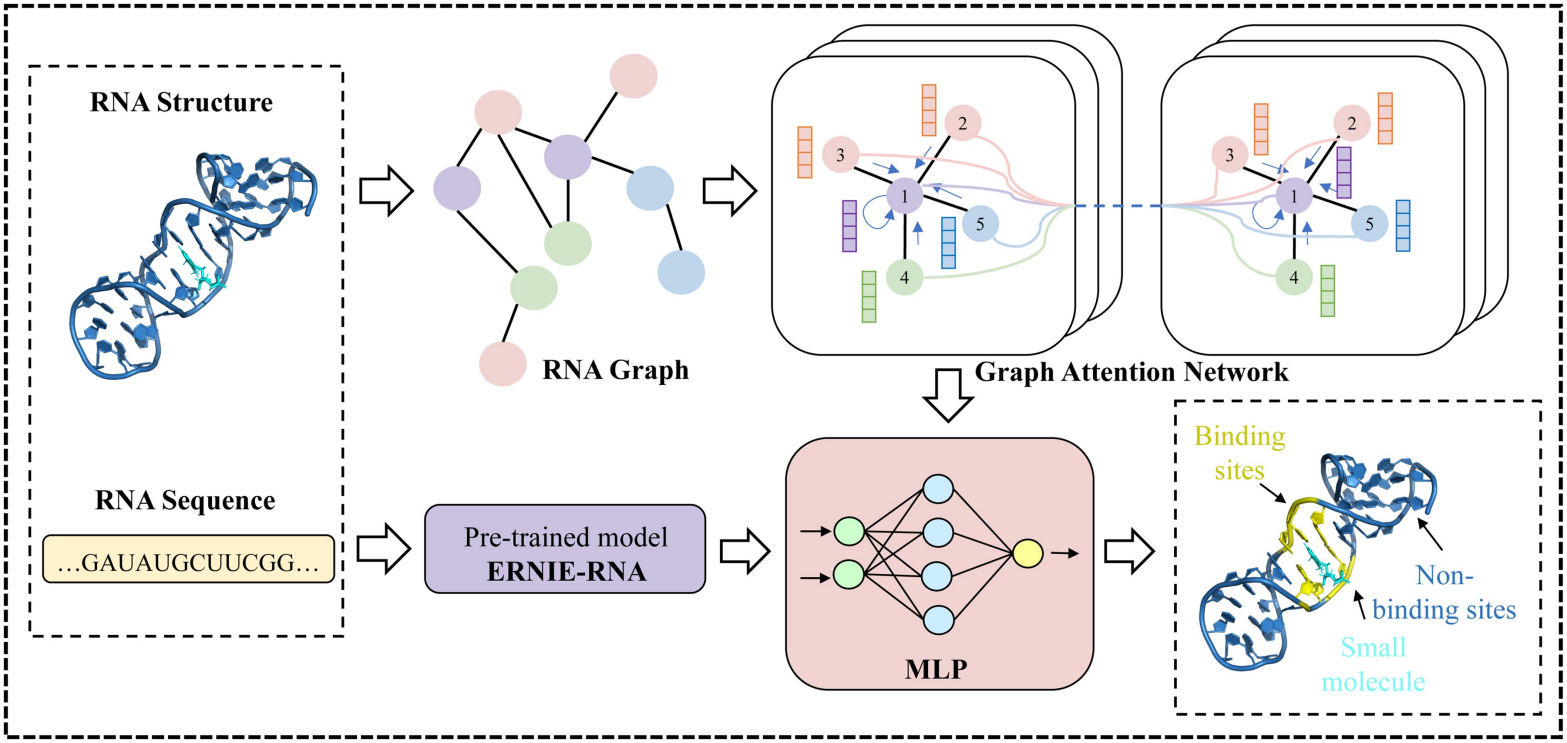
Overview of the RLsite framework. The RNA structure was first represented as a graph and fed into a GAT to extract structural features. In parallel, the corresponding RNA sequence was input into the pre-trained RNA language model (ERNIE-RNA) to capture sequence-based contextual representations. The outputs from both modules were then fused and passed through a Multilayer Perceptron (MLP), which aligns the feature representations. Finally, the classification layer of the MLP predicted the RNA-small molecule binding sites.

To effectively harness the extensive RNA sequence data, we integrated a pre-trained RNA language model (ERNIE-RNA) and a GAT to process RNA sequences and RNA structure-based graphs, respectively. The ERNIE-RNA encodes contextualized sequential representations from large-scale RNA sequences to capture binding information and intrinsic sequence patterns, while the GAT operates on graph representations of RNA static tertiary structures to extract spatial and structural features that contribute to the accurate identification of small-molecule binding sites. After these two parallel modules independently process their inputs, their output feature vectors, representing structural and sequential information respectively, are concatenated and passed into a Multilayer Perceptron (MLP). The MLP serves a crucial role in coupling the different modalities: feature fusion, dimensional alignment, and binding site prediction. The overall framework of our proposed model is depicted in Fig. 1.

### 2.3 Pre-trained RNA language model

To effectively capture RNA sequence features, we propose to use pre-trained RNA language models. Currently, several large-scale RNA language models are available, including RNA-FM (Chen *et al.* 2022), RNAMSM (Zhang *et al.* 2024), ERNIE-RNA (Yin *et al.* 2024), and RNAErnie ([Bibr btaf447-B40][Bibr btaf447-B40]). Based on a comprehensive evaluation presented in a recent review, ERNIE-RNA was selected as our pre-trained RNA language model due to its structure-enhancement, motif-aware masking strategy, relatively compact model size, and strong performance across various downstream tasks (Wang *et al.* 2025). ERNIE-RNA is a pre-trained RNA language model trained on a large-scale dataset comprising 20.4 million non-redundant ncRNA sequences from the RNAcentral database (Consortium 2021, Yin *et al.* 2024). The model processes input sequences by first converting them into a pairwise position matrix, which replaces the bias of the first layer. From the second layer onward, each layer’s bias is dynamically determined by the attention map generated in the preceding layer. Each token in the input sequence is embedded into a 768D vector space through a stack of 12 transformer blocks, with each transformer containing 12 attention heads. The model is trained in a self-supervised manner using Masked Language Modeling (MLM), where approximately 15% of the nucleotide tokens are randomly masked and predicted during training. This approach enables ERNIE-RNA to learn rich representations of RNA sequences, which are subsequently leveraged in our model to enhance small-molecule binding sites prediction.

### 2.4 Graph attention network

GATs, originally proposed by Velickovic *et al.* (Velikovi *et al.* 2017), provide a powerful framework for modeling graph-structured data by leveraging an attention mechanism to assign importance scores to neighboring nodes dynamically. In RNA structure modeling, where nucleotides and their interactions can be naturally represented as graphs, GAT offers a flexible and efficient approach for capturing both local and long-range dependencies within RNA structures.

#### 2.4.1 Graph attention mechanism

Given an RNA molecular graph G=(V,E) , where nodesV represent nucleotides with 11D base-level features and edgesE capture interactions such as Watson-Crick base pairing, non-canonical base pairs, and backbone connectivity from DSSR adjacency annotations (Lu 2020). GAT computes the influence of each neighboring node through an attention mechanism. Unlike traditional graph neural networks that apply uniform weighting to all neighbors, GAT introduces a self-attention mechanism to compute edge-specific attention scores. For each node pair (i,j) connected in the RNA graph, the attention coefficient $e_{ij}$ is calculated as follows:

(1) eij=LeakyReLU(aT[Whi∥Whj])

where:

-
$W\in R^{F^{\prime }\times F}$ is a learnable weight matrix that transforms node features into a new dimension space $F^{\prime }$ ;
-
$a\in R^{2F^{\prime }}$ is a learnable attention weight vector;
-
∥ represents the concatenation of feature vectors;
- LeakyReLU introduces non-linearity, ensuring better gradient flow.

To normalize these scores across different neighbors, a softmax function is applied:

(2) αij= exp(e)ij∑k∈Ni exp(e)ik

where$N_{i}$ denotes the set of neighbors of node i . This normalization ensures that attention scores sum to 1, allowing the model to prioritize critical interactions within the RNA structure.

#### 2.4.2 Feature aggregation and update

Once the attention scores $\alpha_{ij}$ are computed, the node features are updated by aggregating information from their neighbors:

(3) $$h_{i}^{\prime }=\sigma \left(\sum_{j\in N_{i}}\alpha_{ij}Wh_{j}\right)$$

where σ is a non-linear activation function (e.g. ELU or ReLU), and $h_{i}^{\prime }$ represents the updated feature representation of node i . By focusing on the most relevant structural components adaptively, GAT enhances the model’s capacity to identify potential RNA-small molecule binding sites.

#### 2.4.3 Multi-head attention for improved representation

To improve stability and enhance feature representation, GAT uses multi-head attention, where K independent attention heads operate in parallel:

(4) $$h_{i}^{\prime }={∥}_{k=1}^{K}\sigma \left(\sum_{j\in N_{i}}\alpha_{ij}^{k}W^{k}h_{j}\right)$$

Alternatively, an averaging mechanism can be used:

(5) $$h_{i}^{\prime }=\sigma \left(\frac{1}{K}\sum_{k=1}^{K}\sum_{j\in N_{i}}\alpha_{ij}^{k}W^{k}h_{j}\right)$$

where $W^{k}$ and $\alpha_{ij}^{k}$ are the learnable parameters of the k-th attention head. This multi-head mechanism allows the model to capture diverse structural relationships within RNA molecules.

### 2.5 Graph node representations

In our study, three types of node representations were used: (i) Secondary Structures (SS), (ii) Accessible Surface Areas (ASA), and (iii) Torsions (TOR), which capture critical biochemical and structural properties of RNA molecules that directly influence ligand binding.

#### 2.5.1 Secondary structures

SS define the base-pairing and folding motifs of RNA (e.g. stems, loops, bulges), which play a central role in shaping the 3D architecture of RNA. These motifs often create binding pockets or scaffolds that directly interact with small molecules. By encoding these features as node attributes, the model gains contextual awareness of where functionally relevant regions (e.g. aptamer loops or helical stacks) are located. RNA secondary structures were categorized into 11 distinct types, each represented by specific symbols, as shown in Table S2, available as supplementary data at *Bioinformatics* online.

#### 2.5.2 Accessible surface areas

ASA quantifies the extent to which the RNA surface is exposed to solvent molecules, thereby indicating regions potentially accessible to ligand binding. Nucleotides with high ASA are more likely to be accessible for ligand interaction, while buried nucleotides are less so. By incorporating ASA features, the model can learn to differentiate between sterically available and occluded regions, improving binding sites discrimination. Three types of accessible surface areas were calculated, including Total ASA, Polar ASA, and Apolar ASA, reflecting the exposure of RNA atoms to solvent.

#### 2.5.3 Torsions

RNA backbones are highly flexible, and their torsional angles (e.g. alpha, beta, chi) dictate conformational variability. Torsion angles can indicate dynamic or flexible regions, which are often involved in ligand binding due to induced-fit or allosteric mechanisms. By capturing these local dynamics, the model can account for conformational adaptability, which is usually missed in static structural models. A total of 16 torsional angles were extracted to capture conformational flexibility, including Alpha, Beta, Gamma, Delta, Epsilon, Zeta, Epsilon_Zeta, Chi, Splay_angle, Eta, Theta, Eta_prime, Theta_prime, Eta_base, Theta_base, and Phase_angle.

The secondary structures and torsions were derived from RNA tertiary structures using DSSR (Lu 2020), and ASA values were computed using FreeSASA (Mitternacht 2016). These node-level features are crucial for encoding the RNA into a graph-based format suitable for our model. RNA possesses highly complex structures, including diverse bases, base pairs, and chain configurations. Utilizing these structural features is highly beneficial for predicting RNA-small molecule binding sites. To evaluate the contribution of each representation type, we performed a feature ablation analysis, as discussed in Section 3.2.

### 2.6 Feature fusion and prediction module

The extracted features from GAT and ERNIE-RNA are concatenated and passed through a Multilayer Perceptron (MLP). This MLP performs two roles: feature alignment and binding site prediction. It ensures uniform dimensionality and semantic alignment of representations from different modalities. The final classification layer outputs probabilities for each nucleotide, indicating whether it is part of a binding site. This late fusion strategy enables the model to integrate contextual, spatial, and chemical cues for precise site-level prediction.

### 2.7 Performance evaluation

In this work, to evaluate the performance of our method and compare it with other baseline methods, we adopted the widely used evaluation metrics in the protein-ligand binding sites prediction task, including Precision, Recall, Matthews Correlation Coefficient (MCC), and Area Under Curve (AUC). The AUC is a performance metric commonly used in binary classification tasks, especially for evaluating models in imbalanced datasets. AUC is derived from the Receiver Operating Characteristic (ROC) curve, which plots the True Positive Rate (TPR, or Recall) against the False Positive Rate (FPR) at various threshold settings. The definitions of the other three metrics are as follows:

$$\mathrm{Precision}=\frac{TP}{TP+FP}$$

 

$$\mathrm{Recall}=\frac{TP}{TP+FN}$$

 

$$\mathrm{MCC}=\frac{TP\times TN-FP\times FN}{\sqrt{\left(TP+FP)(TP+FN)(TN+FP)(TN+FN\right)}}$$

## 3 Results and discussions

### 3.1 Comparison to other methods

To evaluate our model’s performance, we compared our method RLsite with the other six methods on T18 and T3, including Rsite2, RBind, RNAsite, RLBind, RNet, CapBind, MultiModRLBP, and RNABind. As shown in Table 1, our model achieves the highest performance across all evaluation metrics, with a Precision of 0.749, a Recall of 0.654, an MCC of 0.474, and an AUC of 0.828, surpassing all competing methods. Notably, compared to CapBind, which was previously the best-performing model, our approach demonstrates a substantial improvement in precision (0.749 versus0.710), recall (0.654 versus 0.638), MCC (0.474 versus 0.400), and AUC (0.828 versus 0.770), indicating a superior ability to accurately identify binding sites while maintaining robustness. In addition, Furthermore, our model outperforms RLBind and RNAsite, particularly in recall and AUC, showcasing the advantages of integrating a pre-trained RNA language model and GAT for binding sites prediction. These results validate the effectiveness of our approach and underscore its potential for advancing RNA-small molecule interaction studies.

**Table 1.** Performance of different methods on the dataset T18.^a^

Models	Precision	Recall	MCC	AUC
Rsite2	0.370	0.214	0.010	0.474
RBind	0.655	0.173	0.187	0.559
RNAsite	0.675	0.263	0.253	0.776
RLBind	0.681	0.345	0.324	0.720
RNet	0.701	0.357	0.307	–
RNABind	0.126	0.105	0.041	0.471
CapBind	0.710	0.638	0.400	0.770
MultiModRLBP	0.644	0.523	0.378	0.780
RLsite	**0.749**	**0.654**	**0.474**	**0.828**

a All results for the other methods (Rsite2, RBind, RNAsite, RLBind, RNet, CapBind, and MultiModRLBP) were obtained from their respective publications. The results for RNABind were obtained by executing predictions through its web server. The highest value of each metric from different methods is highlighted in bold type.

Additionally, Table 2 presents the results of our method and the other six methods on T3. Our method achieves the highest scores across all evaluation metrics, with a Precision of 0.705, a Recall of 0.706, an MCC of 0.636, and an AUC of 0.902, outperforming all baseline methods. Notably, our method outperformed MultiModRLBP, the second-best method, by a significant margin in precision (+0.205), MCC (+0.175), and AUC (+0.059), indicating its robustness on different data. Compared to RLBind and RNAsite, our approach maintains a more balanced trade-off between precision and recall while significantly improving MCC and AUC. These results further confirm that the pre-trained RNA language model and GAT could enhance the model’s ability to generalize across different datasets, reinforcing its robustness.

**Table 2.** Performance of different methods on the dataset T3.^a^

Methods	Precision	Recall	MCC	AUC
Rsite2	0.303	0.192	0.085	0.342
RBind	0.269	0.269	0.320	0.585
RNAsite	0.173	0.173	0.197	0.686
RLBind	0.519	0.519	0.421	0.783
RNABind	0.00	0.00	0.00	0.500
MultiModRLBP	0.500	**0.712**	0.461	0.843
RLsite	**0.705**	0.706	**0.636**	**0.902**

a All results for the other methods (Rsite2, RBind, RNAsite, RLBind, and MultiModRLBP) were obtained from their respective publications. The results for RNABind were generated by running its web server. Results for RNet and CapBind were unavailable. The highest value of each metric from different methods is highlighted in bold type.

### 3.2 Secondary structure emerges as the dominant feature for RNA binding site prediction

**Figure 2.**
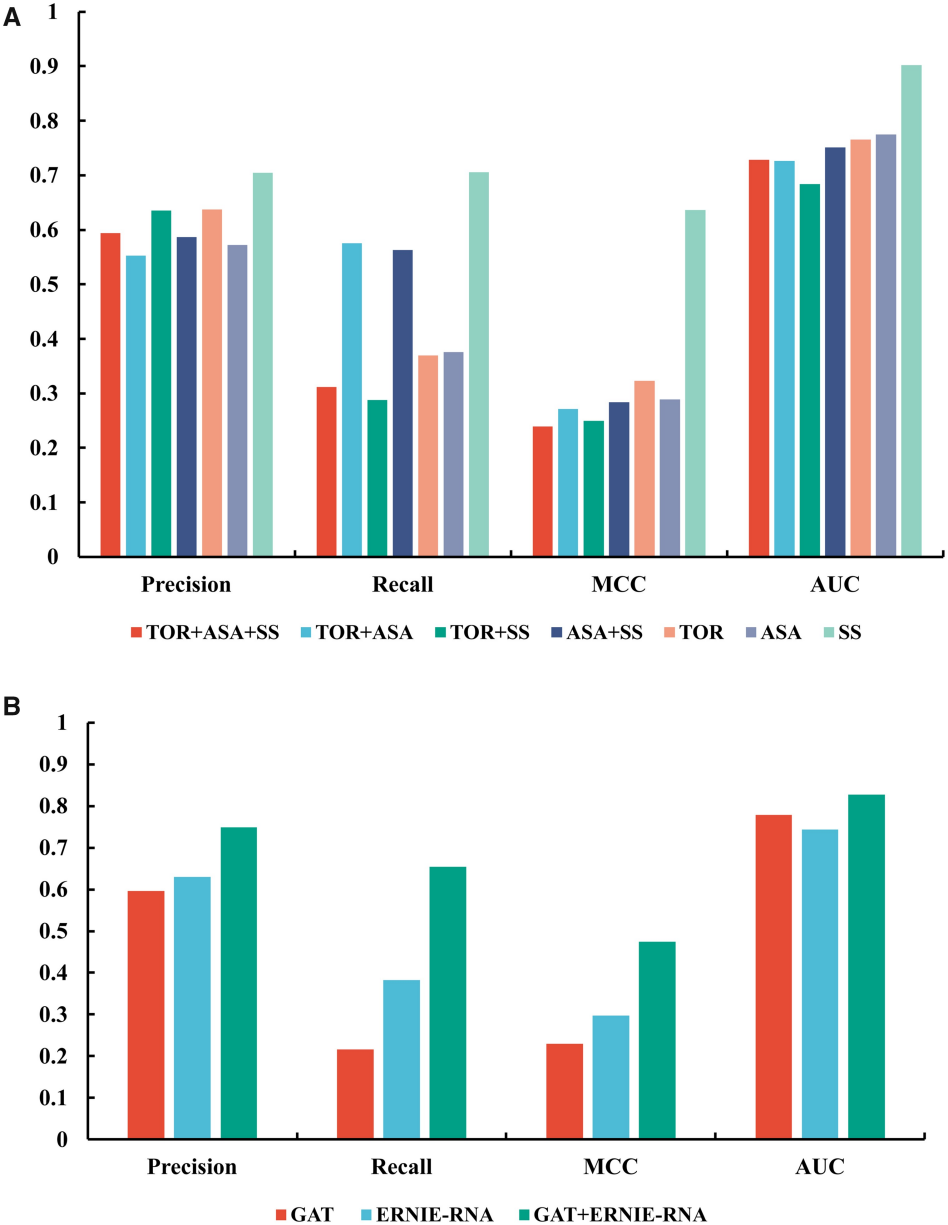
Performance of different features (A) and different modules (B) on T18.

To evaluate the contribution of different RNA structural features to binding site prediction, we performed a feature ablation study on the T18 dataset. Specifically, we assessed the model’s performance when using only torsion angles (TOR), accessible surface area (ASA), secondary structure (SS), or various combinations of these features as input. As illustrated in Fig. 2A, using only secondary structure (SS) yielded the best performance, with a precision of 0.705, recall of 0.706, MCC of 0.636, and AUC of 0.902, suggesting that SS is the most critical feature for accurate binding site prediction. In contrast, models using only torsion (TOR) or ASA features achieved lower performance, with precisions of 0.637 and 0.572, recalls of 0.369 and 0.376, MCCs of 0.323 and 0.289, and AUCs of 0.766 and 0.775, respectively. Interestingly, combining these features resulted in poorer performance compared to using one type of feature alone. This may be attributed to potential redundancy between ASA and SS features, as well as the possibility that torsion features introduce interference information rather than complementary information.

**Figure 3.**
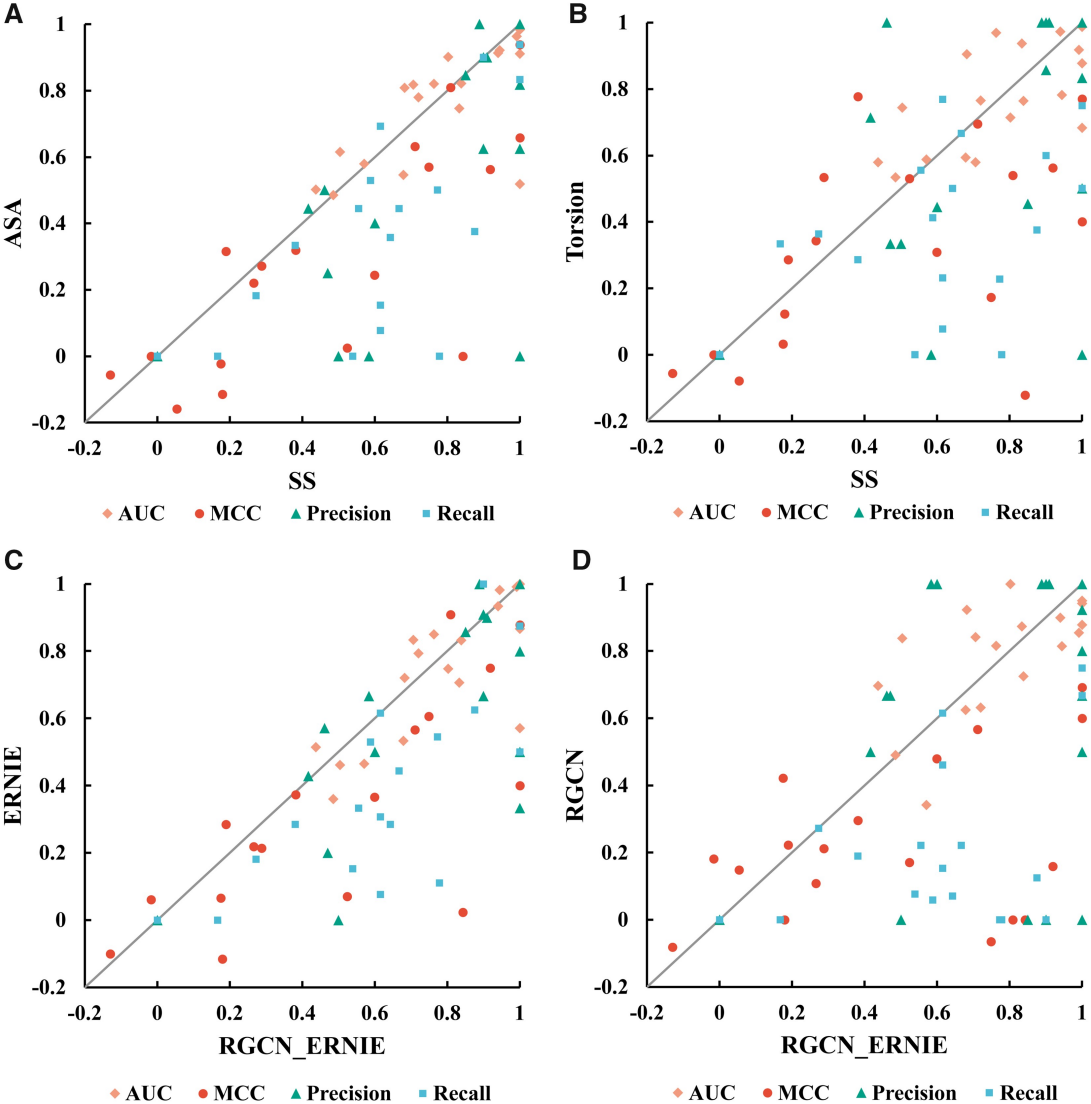
One-to-one comparison between features (A and B) and between modules (C and D) on T18.

Furthermore, we conducted one-to-one comparisons of performance from different features on T18. As shown in Fig. 3A and B, most RNAs in T18 achieved better performance when using SS instead of ASA/TOR as the feature. Very few RNAs have higher metric values (AUCs, MCCs, Precisions, Recalls) when using ASA/TOR as the feature instead of SS as the feature. Specifically, most MCCs, Precisions, and Recalls from SS outperformed the metrics from ASA/TOR remarkably. Besides, most AUCs achieved similar values, and a few AUC values from SS are markedly higher than AUC values from ASA/TOR. These results suggest that secondary structure is dominant in understanding RNA binding properties, while torsion angles and accessible surface area contribute to a lesser extent. Additionally, a combination of these three structural features was attempted as well, but showed less performance than only SS.

These node-level features collectively enable our model to: understand local biochemical environments at the residue level, infer structural-functional relationships tied to ligand recognition, and simulate a level of conformational flexibility (through torsion patterns and ASA exposure) even without explicit time-series or ensemble data. By embedding this biologically rich information into the graph structure, our GAT-based model becomes more context-aware and biophysically grounded, allowing it to generalize better across different RNAs and ligand types compared to models that use only primary sequences or static structural features.

### 3.3 Complementarity of graph attention networks and RNA language models

To further investigate the contribution of each component in our model, we conducted an ablation study on T18 by evaluating the performance of the model using only GAT, only ERNIE-RNA, and the combination of both. As shown in Fig. 2B, integrating GAT and ERNIE-RNA achieves the best performance across all metrics, with a Precision of 0.749, a Recall of 0.654, an MCC of 0.474, and an AUC of 0.828. When using only GAT, the model achieves a lower precision (0.596), recall (0.216), and MCC (0.229), indicating that less structural information alone is insufficient for accurate binding site prediction. On the other hand, using only ERNIE-RNA improves recall (0.382) and MCC (0.297) compared to GAT alone, suggesting that pre-trained sequence representations contribute more significantly to identifying potential binding sites. However, the best performance is achieved when combining both GAT and ERNIE-RNA, highlighting the complementary nature of structural and sequence-based information. This experiment confirms that incorporating attention-graph-based structural learning and pre-trained language model embeddings leads to a more comprehensive and effective RNA-small molecule binding site prediction model.

Specifically, a one-to-one comparison of the performances of different modules on T18 was conducted. Figure 3 presents the metrics comparison of our method with both GAT and ERNIE-RNA modules with our method without the GAT module (C) or the ERNIE-RNA module (D). As can be seen, most RNAs achieved higher performance when using both modules than when using only one. In addition, by comparing Fig. 3C and D, it can be inferred that the GAT module outperformed the ERNIE-RNA module slightly for several RNAs. This illustrates that attention-graph-based structural information is the key to RNA-small molecule binding site prediction when the corresponding sequential information is insufficient.

### 3.4 Consistent high performance of RLsite on the latest dataset T10

To further evaluate the generalization ability of our method, we compared our model’s performance with MultiModRLBP on our newly constructed test set T10. It can be observed that MultiModRLBP and CapBind achieved comparably high performance, albeit slightly lower than that of RLsite. Therefore, due to the unavailability of CapBind, we restricted our comparison to MultiModRLBP and our proposed method. As shown in Table 3, our method significantly outperforms MultiModRLBP in recall (0.596 versus 0.164) and MCC (0.343 versus 0.161). Additionally, our model achieves a higher AUC (0.717 versus 0.684), indicating improved overall classification performance. While MultiModRLBP has a slightly higher precision (0.471 versus 0.463), its lower recall suggests a tendency to miss many native binding sites. These results highlight the effectiveness of our approach in predicting RNA-small molecule binding sites, particularly in improving sensitivity while maintaining competitive precision.

**Table 3.** Additional test on T10 of MultiModRLBP and RLsite.

Methods	Precision	Recall	MCC	AUC
MultiModRLBP	**0.471**	0.164	0.161	0.684
RLsite	0.463	**0.596**	**0.343**	**0.717**

a The highest value of each metric from different methods is highlighted in bold type.

**Figure 4.**
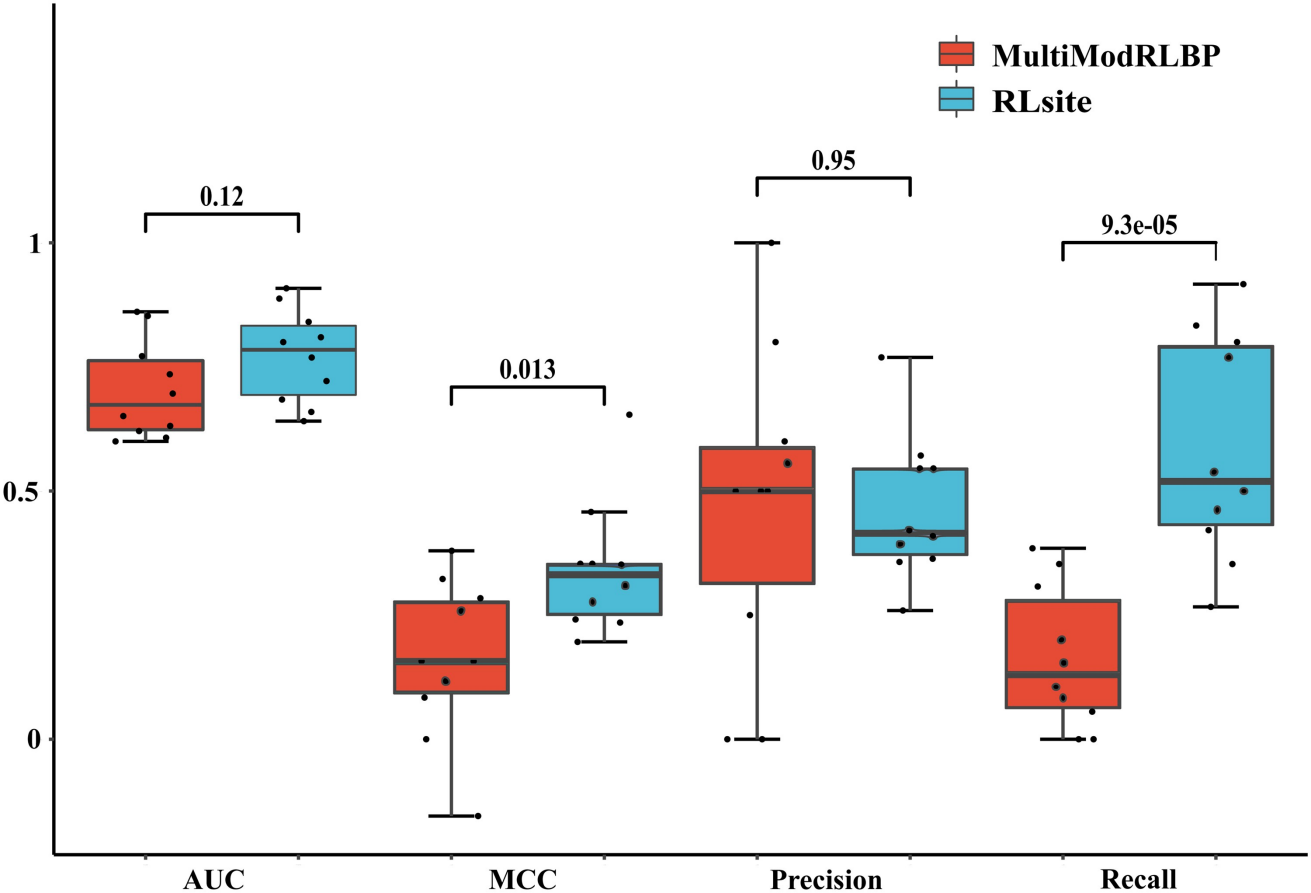
The metrics distribution of MultiModRLBP and RLsite on T10.

To further analyze the performance differences between MultiModRLBP and our proposed method RLsite, we examined the distributions of various evaluation metrics (AUC, MCC, Precision, and Recall) across all RNA samples in T10. Figure 4 presents box plots for each metric, offering a more detailed comparison between the two models. As observed, the mean values align with the results in Table 3, confirming that RLsite achieves a higher AUC, while MultiModRLBP has a slightly higher Precision. However, RLsite achieves significantly higher Recall and MCC compared to MultiModRLBP, indicating that despite having similar precision values, MultiModRLBP could only predict fewer binding sites than RLsite. Furthermore, the precision values of MultiModRLBP show more significant variability, with more instances exhibiting notably lower precision scores. This suggests that MultiModRLBP achieves a higher average precision but lacks consistency across different RNA samples.

Furthermore, t-tests were also conducted to assess the statistical significance of all metrics. The results indicate no significant difference between MultiModRLBP and RLsite in the AUC and Precision groups. However, significant differences were observed in MCC and Recall, highlighting that RLsite significantly outperforms MultiModRLBP in detecting RNA-small molecule binding sites, particularly under unbalanced data conditions.

### 3.5 Case studies

**Figure 5.**
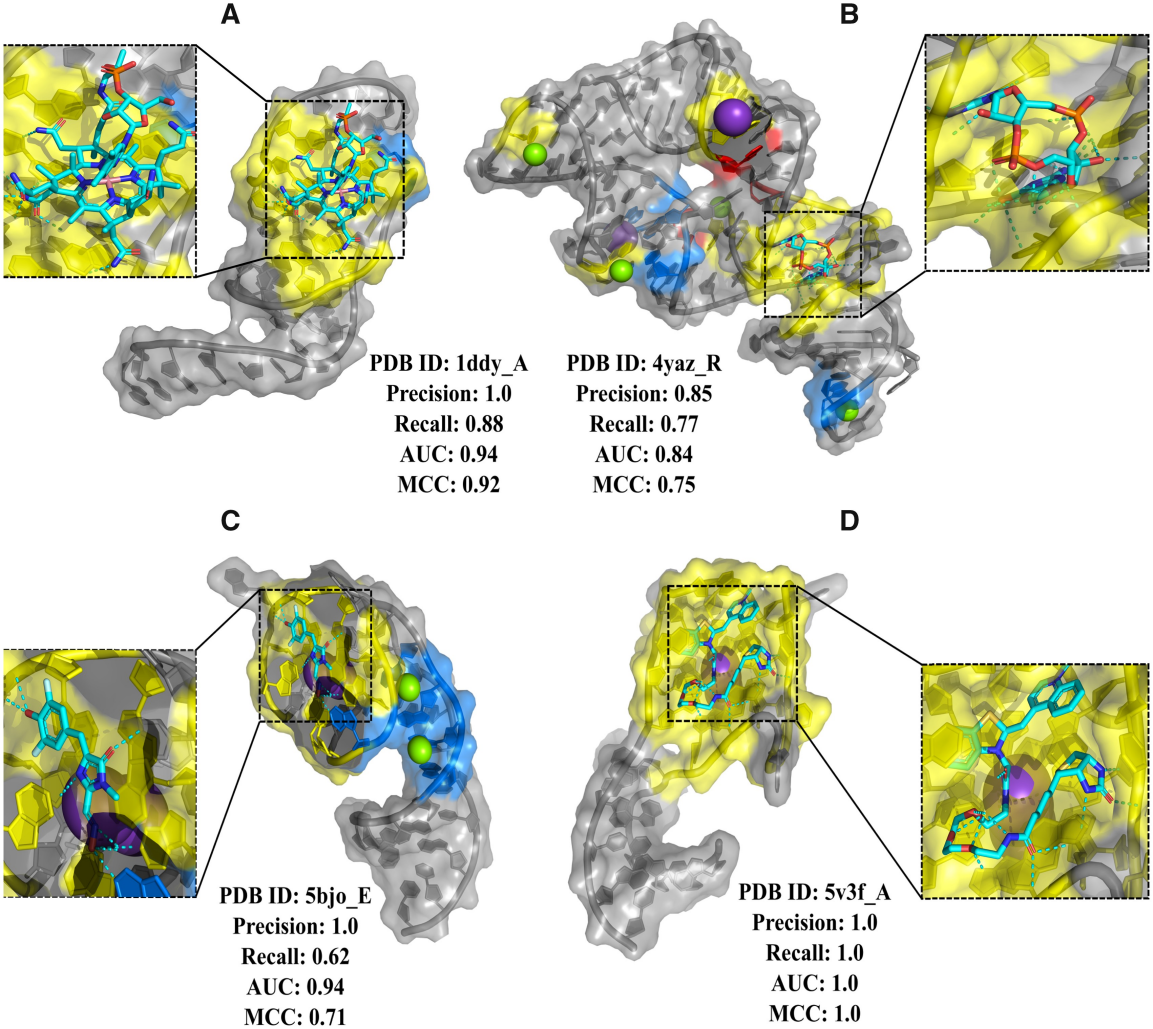
Representative examples of RNA-small molecule interactions. RNA chains are depicted in cartoon and surface mode (gray), while small molecules are shown as stick representations (cyan). Correctly predicted binding sites are highlighted in yellow, misclassified binding sites are in red, and missed binding sites are in blue. (A) RNA interaction with vitamin B12 (PDB ID: 1ddy_A), where a locally folded RNA triplex forms a binding cleft. (B) A riboswitch bound to 3',3'-cGAMP (PDB ID: 4yaz_R) with a junctional ligand-binding pocket. (C) A Corn RNA aptamer in complex with DFHO (PDB ID: 5bjo_E), where the ligand is encapsulated at the interprotomer interface. (D) A fluorogenic RNA Mango complexed with a TO-derived fluorophore (PDB ID: 5v3f_A), where the ligand interacts with a three-tiered G-quadruplex.

To further evaluate the performance of our method, we analyzed four RNA-small molecule interaction complexes from T18, considering RNA types, structural flexibility, and ligand characteristics. Figure 5A presents the interaction between an RNA molecule and vitamin B12 (PDB ID: 1ddy_A). The structure features a locally folding RNA triplex, stabilized by a novel three-stranded zipper. A perpendicular duplex stacking on this triplex creates a cleft that serves as the vitamin B12 binding site (Sussman *et al.* 2000, Johnson *et al.* 2012, Musiari *et al.* 2024). Our method correctly predicted all binding sites within the cleft (highlighted in yellow), with only one misclassified site (C13, highlighted in blue), which was located at the end of a duplex slightly farther from the cleft. Hence, 87.5% of the binding sites were predicted accurately. Figure 5B shows a riboswitch bound to 3',3'-cGAMP (PDB ID: 4yaz_R). This riboswitch adopts a tuning fork-like architecture with a junctional ligand-binding pocket. The binding orientation of the arms is influenced by the identity of the bound cyclic dinucleotide (Ren *et al.* 2015, Li *et al.* 2018). Our method successfully identified most 3',3'-cGAMP binding sites (yellow) but exhibited lower accuracy in predicting metal ion (Mg^2+^ and K^+^) binding sites. Some binding sites near Mg^2+^ and K^+^ were missed (blue), while others around a different Mg^2+^ ion were incorrectly classified as binding sites (red). Figure 5C presents a Corn RNA aptamer complexed with DFHO (PDB ID: 5bjo_E). The Corn-DFHO co-crystal structure revealed that its functional species is a quasi-symmetric homodimer, encapsulating one DFHO molecule at the interprotomer interface, sandwiched between two G-quadruplexes (Warner *et al.* 2017, Sjekloca and Ferre-D'Amare 2019, Bereiter *et al.* 2025). As shown in Fig. 5C, our method accurately predicted most binding sites near DFHO, yet, similar to Fig. 5B, it failed to identify binding sites around Mg^2+^ and K^+^ ions. Figure 5D demonstrates a fluorogenic RNA Mango bound to a thiazole orange (TO)-derived fluorophore (PDB ID: 5v3f_A). RNA Mango exhibits an exceptionally high affinity for TO1-Biotin (Kd ∼3 nM) and, in complex with related ligands, represents one of the most redshifted fluorescent macromolecular tags known. In this structure, the entire ligand, including TO, biotin, and its linker, is positioned adjacent to a three-tiered G-quadruplex, with the two heterocycles of TO stabilized by loop adenines at a 45° angle (Dolgosheina *et al.* 2014, Trachman *et al.* 2017, Trachman *et al.* 2019). Unlike previous cases, this structure lacked metal ions, resulting in higher accuracy in binding site detection.

These case studies demonstrate that our method performs well in predicting binding sites for non-metal small molecules but exhibits reduced performance in detecting metal ion interactions. Our method likely does not explicitly account for metal coordination chemistry, such as electrostatic interactions, charge density, or coordination geometry (e.g. octahedral, tetrahedral), which are essential for metal ion binding. Besides, Metal ions often bind through short-range electrostatic interactions or coordinate with negatively charged atoms (e.g. phosphate oxygens), which are not explicitly modeled in sequence embeddings or graph node attributes. Metal ion-RNA interactions are underrepresented in many structural datasets. As a result, the model may not be exposed to enough positive examples during training, limiting its ability to generalize to these cases. Binding of metal ions can stabilize specific RNA conformations or induce subtle rearrangements. A static structural snapshot may miss these conformational nuances, leading to poor prediction of binding regions. Most existing models, including RBind, RNAsite, RLBind, and MultiModRLBP, primarily focus on organic small molecules and do not perform well on metal ions, largely due to similar limitations: lack of electrostatics, coordination features, and sparse data on metal-bound RNA complexes. This limitation is well-documented in the literature but is often under-emphasized due to the broader focus on organic ligand interactions. This highlights the need for further design in handling metal-coordinated RNA-ligand interactions.

### 3.6 Potential drug binding sites

**Figure 6.**
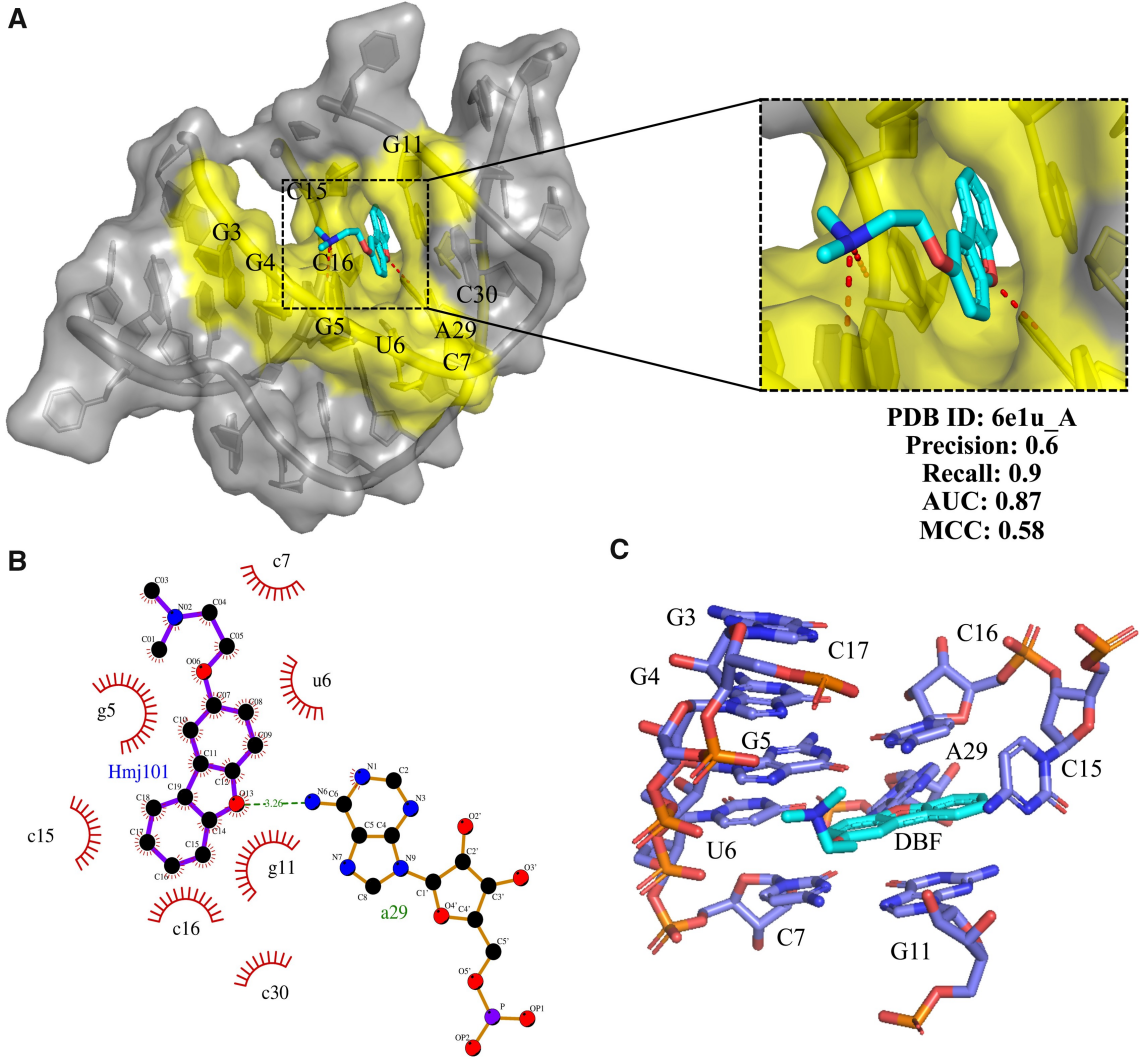
Validation of predicted binding sites for the PreQ1 Riboswitch (PDB ID: 6e1u_A). (A) 3D structural representation of the PreQ1 riboswitch complex visualized using PyMOL, where the RNA chain is shown in gray, the native binding sites in yellow, and the small molecule ligand in cyan. (B) The 2D visualization of the interactions generated by LigPlot+ illustrates hydrophobic interactions (red short lines) and hydrogen bonds (green lines) between nucleotides and the ligand (Laskowski and Swindells 2011). (C) Experimentally identified drug-like small molecules (DBF) that noncovalently bind to the PreQ1 riboswitch, discovered through screening strategies in previous studies.

To further assess the effectiveness of our approach, we validated it on a PreQ1 riboswitch (PDB ID: 6e1u_A). PreQ1 is a metabolic intermediate in the biosynthesis of queuosine (Q), a hypermodified guanine nucleotide that is widespread across eubacteria and eukaryotes (Grosjean *et al.* 2004, Díaz-Rullo and González-Pastor 2023). Notably, reduced levels of Q-modified tRNA (Q—tRNA) have been associated with neoplastic transformation and proposed as a potential biomarker for tumor grading (Huang *et al.* 1992, Díaz-Rullo *et al.* 2025). Figure 6A presents the crystal structure of a class I PreQ1 riboswitch complexed with the synthetic compound 2-[(dibenzo[b, d]furan-2-yl)oxy]-N, N-dimethylethan-1-amine, along with its interaction details. Our method successfully identified most of the binding sites (G3, G4, G5, U6, C7, G11, C16, A29, and C30), missing only one site (C15). The model achieved a Precision of 0.6, a Recall of 0.9, an AUC of 0.97, and an MCC of 0.58, demonstrating strong predictive performance. Figure 6B provides a 2D interaction diagram, where binding sites were determined based on hydrophobic interactions and hydrogen bonds rather than simple Euclidean distances between nucleotides and small molecules. Notably, our predicted binding sites align with experimental validations by Balaratnam, S. *et al.* (Balaratnam *et al.* 2021), who engineered covalent small molecule-RNA complexes in living cells. Figure 6C exhibits an experimentally identified micromolar dibenzofuran binder, which was discovered through high-throughput screening strategies (Bereiter *et al.* 2025). This small molecule shares the same binding sites as the ligand in Fig. 5A, except for C17, which was correctly predicted as a binding site by our method. This result demonstrates the accuracy and robustness of our approach in predicting RNA-ligand binding sites across different ligands. These findings confirm that our model’s predictions are biologically relevant and supported by experimental data, highlighting the potential of our method in gene expression regulation and RNA-targeted drug discovery.

## 4 Conclusions

In this study, we proposed RLsite, a novel deep learning framework that integrates a pre-trained RNA language model (ERNIE-RNA) with a GAT to accurately predict small-molecule binding sites on RNA. By leveraging both sequence-level information from large-scale RNA datasets and structural features derived from RNA tertiary structures, our approach addresses several key limitations of existing methods, such as limited data availability, insufficient structure information transmission, and lack of biological validation.

We demonstrated that combining node-level representations, such as secondary structures, accessible surface areas, and torsional angles, with the contextual embeddings from a pre-trained language model enables RLsite to capture the complex biochemical and spatial features critical for RNA-ligand interactions. The model outperformed traditional machine learning and recent deep learning approaches across benchmark datasets and maintained high predictive accuracy and sensitivity in a real-world case study of the PreQ1 riboswitch (PDB ID: 6e1u_A), with predictions strongly aligned with experimental observations. Importantly, RLsite also successfully predicted binding sites consistent across different ligands, highlighting its robustness and generalizability. These findings validate the model’s utility for computational prediction and as a tool to support RNA-targeted drug discovery and the functional annotation of RNA structures.

RLsite outperforms other methods primarily due to its innovative integration of sequence-based and structure-based information, which allows it to capture RNA-ligand interactions more comprehensively than previous models. Here are the key points for its superior performance:

### 4.1 Hybrid architecture leveraging complementary modalities

GAT: RLsite uses a GAT to model RNA tertiary structures as graphs, which enables the model to effectively learn spatial and topological dependencies among nucleotides. This is particularly important for capturing the 3D context of binding sites.

Pretrained RNA Language Model (ERNIE-RNA): The use of a large-scale pretrained RNA language model allows RLsite to embed RNA sequences with contextual information learned from millions of natural RNA sequences. This helps capture functional and evolutionary patterns that may not be evident from structure alone.

### 4.2 Biologically informed node representations

RLsite encodes each RNA nucleotide using a multi-view feature representation. These representations provide rich biochemical and geometric context, improving the model’s ability to localize plausible binding sites. Prior models such as RLBind or CapBind may not incorporate such detailed, low-level structural descriptors, leading to less precise binding site localization.

### 4.3 Better handling of limited structural data

Through the use of pretrained language models, RLsite compensates for the scarcity of tertiary structure data. This gives it an advantage over structure-only models (e.g. Rsite) and earlier machine learning models that lacked deep sequence context.

Despite notable advancements, existing methods for RNA-small molecule binding site prediction face several limitations. Many rely on static structural representations and fail to account for the dynamic and flexible nature of RNA conformations, leading to reduced predictive accuracy. Most methods suffer from limited generalizability due to small or biased training datasets and often lack integration of diverse biological features. Furthermore, few models effectively incorporate experimental validation, which restricts their translational applicability. In future work, integrating conformational ensemble modeling, expanding training datasets with experimentally verified interactions, and incorporating additional features, such as metal ion coordination, solvent effects, or RNA modifications, may enhance model accuracy and robustness. Combining experimental feedback with computational predictions could also bridge the gap between in silico analysis and practical drug discovery applications. Future work will also explore integrating experimental high-throughput binding data to refine the model’s training and validate its applicability in broader biological contexts. Altogether, RLsite represents a significant step forward in the computational exploration of RNA-small molecule interactions and offers a promising avenue for structure-informed RNA therapeutic development.

## Supplementary Material

btaf447_Supplementary_Data

## Data Availability

The resource codes and data of RLsite are accessible at https://github.com/SaisaiSun/RLsite.

## References

[btaf447-B1] Balaratnam S , Rhodes C, Bume DD et al A chemical probe based on the PreQ(1) metabolite enables transcriptome-wide mapping of binding sites. Nat Commun 2021;12:5856. 34615874 10.1038/s41467-021-25973-x PMC8494917

[btaf447-B2] Bereiter R , Flemmich L, Nykiel K et al Engineering covalent small molecule-RNA complexes in living cells. Nat Chem Biol 2025;21:843–54. 39762536 10.1038/s41589-024-01801-3 PMC12122380

[btaf447-B3] Burley SK , Bhatt R, Bhikadiya C et al Updated resources for exploring experimentally-determined PDB structures and computed structure models at the RCSB protein data bank. Nucleic Acids Res 2025;53:D564–74. 39607707 10.1093/nar/gkae1091 PMC11701563

[btaf447-B4] Chen J, Hu Z, Sun S et al Interpretable RNA foundation model from unannotated data for highly accurate RNA structure and function predictions. arXiv, arXiv:2204.00300, 2022, preprint: not peer reviewed.

[btaf447-B5] Chen S , Mao Q, Cheng H et al RNA-Binding small molecules in drug discovery and delivery: an overview from fundamentals. J Med Chem 2024;67:16002–17. 39287926 10.1021/acs.jmedchem.4c01330

[btaf447-B6] Childs-Disney JL , Yang X, Gibaut QMR et al Targeting RNA structures with small molecules. Nat Rev Drug Discov 2022;21:736–62. 35941229 10.1038/s41573-022-00521-4 PMC9360655

[btaf447-B7] Coan M , Haefliger S, Ounzain S et al Targeting and engineering long non-coding RNAs for cancer therapy. Nat Rev Genet 2024;25:578–95. 38424237 10.1038/s41576-024-00693-2

[btaf447-B8] Consortium, R.N RNAcentral 2021: secondary structure integration, improved sequence search and new member databases. Nucleic Acids Res 2021;49:D212–20. 33106848 10.1093/nar/gkaa921 PMC7779037

[btaf447-B9] Díaz-Rullo J , González-Pastor JE. tRNA queuosine modification is involved in biofilm formation and virulence in bacteria. Nucleic Acids Res 2023;51:9821–37. 37638766 10.1093/nar/gkad667 PMC10570037

[btaf447-B10] Díaz-Rullo J , González-Moreno L, Del Arco A et al Decoding the general role of tRNA queuosine modification in eukaryotes. Sci Rep 2025;15:345. 39747999 10.1038/s41598-024-83451-y PMC11695743

[btaf447-B11] Dolgosheina EV , Jeng SCY, Panchapakesan SSS et al RNA mango aptamer-fluorophore: a bright, high-affinity complex for RNA labeling and tracking. ACS Chem Biol 2014;9:2412–20. 25101481 10.1021/cb500499x

[btaf447-B12] Ganser LR , Lee J, Rangadurai A et al High-performance virtual screening by targeting a high-resolution RNA dynamic ensemble. Nat Struct Mol Biol 2018;25:425–34. 29728655 10.1038/s41594-018-0062-4 PMC5942591

[btaf447-B13] Ghidini A , Cléry A, Halloy F et al RNA-PROTACs: degraders of RNA-binding proteins. Angew Chem Int Ed Engl 2021;60:3163–9. 33108679 10.1002/anie.202012330 PMC7898822

[btaf447-B14] Grosjean H , de Crécy-Lagard V, Björk GR. Aminoacylation of the anticodon stem by a tRNA-synthetase paralog: relic of an ancient code? Trends Biochem Sci 2004;29:519–22. 15450604 10.1016/j.tibs.2004.08.005

[btaf447-B15] He S , Valkov E, Cheloufi S et al The nexus between RNA-binding proteins and their effectors. Nat Rev Genet 2023;24:276–94. 36418462 10.1038/s41576-022-00550-0 PMC10714665

[btaf447-B16] Huang BS , Wu RT, Chien KY. Relationship of the queuine content of transfer ribonucleic acids to histopathological grading and survival in human lung cancer. Cancer Res 1992;52:4696–700. 1511436

[btaf447-B17] Johnson JE , Reyes FE, Polaski JT Jr., et al B12 cofactors directly stabilize an mRNA regulatory switch. Nature 2012;492:133–7. 23064232 10.1038/nature11607 PMC3518761

[btaf447-B18] Jung V , Vincent-Cuaz C, Tumescheit C et al Decoding the interactions and functions of non-coding RNA with artificial intelligence. Nat Rev Mol Cell Biol 2025;9:1–22. 10.1038/s41580-025-00857-w 40537558

[btaf447-B19] Laskowski RA , Swindells MB. LigPlot+: multiple ligand-protein interaction diagrams for drug discovery. J Chem Inf Model 2011;51:2778–86. 21919503 10.1021/ci200227u

[btaf447-B20] Li C , Zhao X, Zhu X et al Structural studies of the 3',3'-cGAMP riboswitch induced by cognate and noncognate ligands using molecular dynamics simulation. Int J Mol Sci 2018;19:3527. 30423927 10.3390/ijms19113527 PMC6274999

[btaf447-B21] Liu B. BioSeq-Analysis: a platform for DNA, RNA and protein sequence analysis based on machine learning approaches. Brief Bioinform 2019;20:1280–94. 29272359 10.1093/bib/bbx165

[btaf447-B22] Liu H , Jian Y, Hou J et al RNet: a network strategy to predict RNA binding preferences. Brief Bioinform 2024;25:1–13. 10.1093/bib/bbad482 PMC10749790 38145947

[btaf447-B23] Lu XJ. DSSR-enabled innovative schematics of 3D nucleic acid structures with PyMOL. Nucleic Acids Res 2020;48:e74. 32442277 10.1093/nar/gkaa426 PMC7367123

[btaf447-B24] Mallet V , Oliver C, Broadbent J et al RNAglib: a python package for RNA 2.5 D graphs. Bioinformatics 2022;38:1458–9. 34908108 10.1093/bioinformatics/btab844

[btaf447-B25] Mitternacht S. FreeSASA: an open source C library for solvent accessible surface area calculations. F1000Res 2016;5:189. 26973785 10.12688/f1000research.7931.1 PMC4776673

[btaf447-B26] Musiari A et al Corrin ring modifications reveal the chemical and spatial requirements for the B12‐btuB riboswitch interaction. Chem Eur J 2024;30:e202401800. 38922714 10.1002/chem.202401800

[btaf447-B27] Nemeth K , Bayraktar R, Ferracin M et al Non-coding RNAs in disease: from mechanisms to therapeutics. Nat Rev Genet 2024;25:211–32. 37968332 10.1038/s41576-023-00662-1

[btaf447-B28] Panei FP , Gkeka P, Bonomi M. Identifying small-molecules binding sites in RNA conformational ensembles with SHAMAN. Nat Commun 2024;15:5725. 38977675 10.1038/s41467-024-49638-7 PMC11231146

[btaf447-B29] Ren A , Wang XC, Kellenberger CA et al Structural basis for molecular discrimination by a 3',3'-cGAMP sensing riboswitch. Cell Rep 2015;11:1–12. 25818298 10.1016/j.celrep.2015.03.004 PMC4732562

[btaf447-B30] Rizvi NF , Santa Maria JP, Nahvi A et al Targeting RNA with small molecules: identification of selective, RNA-binding small molecules occupying Drug-Like chemical space. SLAS Discov 2020;25:384–96. 31701793 10.1177/2472555219885373

[btaf447-B31] Shao Y , Zhang QC. Targeting RNA structures in diseases with small molecules. Essays Biochem 2020;64:955–66. 33078198 10.1042/EBC20200011 PMC7724634

[btaf447-B32] Sjekloca L , Ferre-D'Amare AR. Binding between G quadruplexes at the homodimer interface of the corn RNA aptamer strongly activates thioflavin T fluorescence. Cell Chem Biol 2019;26:1159–68 e1154. 31178406 10.1016/j.chembiol.2019.04.012 PMC6697623

[btaf447-B33] Su H , Peng Z, Yang J. Recognition of small molecule-RNA binding sites using RNA sequence and structure. Bioinformatics 2021;37:36–42. 33416863 10.1093/bioinformatics/btaa1092 PMC8034527

[btaf447-B34] Sussman D , Nix JC, Wilson C. The structural basis for molecular recognition by the vitamin B 12 RNA aptamer. Nat Struct Biol 2000;7:53–7. 10625428 10.1038/71253

[btaf447-B35] Trachman RJ , Autour A, Jeng SCY 3rd, et al Structure and functional reselection of the Mango-III fluorogenic RNA aptamer. Nat Chem Biol 2019;15:472–9. 30992561 10.1038/s41589-019-0267-9 PMC7380332

[btaf447-B36] Trachman RJ , Demeshkina NA, Lau MWL 3rd, et al Structural basis for high-affinity fluorophore binding and activation by RNA Mango. Nat Chem Biol 2017;13:807–13. 28553947 10.1038/nchembio.2392 PMC5550021

[btaf447-B37] Velikovi P, Cucurull, G, Casanova, A et al *Graph Attention Networks*. *arXiv, arXiv:1710.10903,* 2017, preprint: not peer reviewed.

[btaf447-B38] Wang H, Zhang Y, Chen J et al A comparative review of RNA language models. arXiv, arXiv:2505.09087, 2025, preprint: not peer reviewed.

[btaf447-B39] Wang J , Quan L, Jin Z et al MultiModRLBP: a deep learning approach for multi-modal RNA-small molecule ligand binding sites prediction. IEEE J Biomed Health Inform 2024a;28:4995–5006. 38739505 10.1109/JBHI.2024.3400521

[btaf447-B40] Wang N , Bian J, Li Y et al Multi-purpose RNA language modelling with motif-aware pretraining and type-guided fine-tuning. Nat Mach Intell 2024b;6:548–57.

[btaf447-B41] Wang K , Jian Y, Wang H et al RBind: computational network method to predict RNA binding sites. Bioinformatics 2018;34:3131–6. 29718097 10.1093/bioinformatics/bty345

[btaf447-B42] Wang K , Zhou R, Wu Y et al RLBind: a deep learning method to predict RNA-ligand binding sites. Brief Bioinform 2023a;24:1–12. 10.1093/bib/bbac486 36398911

[btaf447-B43] Wang R , Jiang Y, Jin J et al DeepBIO: an automated and interpretable deep-learning platform for high-throughput biological sequence prediction, functional annotation and visualization analysis. Nucleic Acids Res 2023b;51:3017–29. 36796796 10.1093/nar/gkad055 PMC10123094

[btaf447-B44] Warner KD , Sjekloća L, Song W et al A homodimer interface without base pairs in an RNA mimic of red fluorescent protein. Nat Chem Biol 2017;13:1195–201. 28945234 10.1038/nchembio.2475 PMC5663454

[btaf447-B45] Wei Q , Wang R, Jiang Y et al ConPep: prediction of peptide contact maps with pre-trained biological language model and multi-view feature extracting strategy. Comput Biol Med 2023;167:107631. 37948966 10.1016/j.compbiomed.2023.107631

[btaf447-B46] Xi W , Wang R, Wang L et al An interpretable deep learning model predicts RNA–small molecule binding sites. Fut Gener Comput. Syst 2024;159:557–66.

[btaf447-B47] Yin W, Zhang Z, He L et al ERNIE-RNA: an RNA language model with structure-enhanced representations. bioRxiv, 2024. 10.1101/2024.03.17.585376, preprint: not peer reviewed. PMC12627772 41253752

[btaf447-B48] Yu AM , Choi YH, Tu MJ. RNA drugs and RNA targets for small molecules: principles, progress, and challenges. Pharmacol Rev 2020;72:862–98. 32929000 10.1124/pr.120.019554 PMC7495341

[btaf447-B49] Zeng P , Cui Q. Rsite2: an efficient computational method to predict the functional sites of noncoding RNAs. Sci Rep 2016;6:19016. 26751501 10.1038/srep19016 PMC4707467

[btaf447-B50] Zeng P , Li J, Ma W et al Rsite: a computational method to identify the functional sites of noncoding RNAs. Sci Rep 2015;5:9179. 25776805 10.1038/srep09179 PMC4361870

[btaf447-B51] Zeng X , Wang F, Luo Y et al Deep generative molecular design reshapes drug discovery. Cell Rep Med 2022;3:100794. 36306797 10.1016/j.xcrm.2022.100794 PMC9797947

[btaf447-B52] Zhang Y , Lang M, Jiang J et al Multiple sequence alignment-based RNA language model and its application to structural inference. Nucleic Acids Res 2024;52:e3. 37941140 10.1093/nar/gkad1031 PMC10783488

[btaf447-B53] Zhu W , Ding X, Shen H-B et al Identifying RNA-small molecule binding sites using geometric deep learning with language models. J Mol Biol 2025;437:169010. 39961524 10.1016/j.jmb.2025.169010

[btaf447-B54] Zhuo C , Zeng C, Liu H et al Advances and mechanisms of RNA-Ligand interaction predictions. Life 2025;15:104. 39860045 10.3390/life15010104 PMC11767038

