# Supplementary material for: RNA language model and graph attention network for RNA and small molecule binding sites prediction
Source: Bioinformatics. 2025 Aug 6;41(9):btaf447. doi: 10.1093/bioinformatics/btaf447 (PMC12417085; doi:10.1093/bioinformatics/btaf447)
Supplement: btaf447_Supplementary_Data [file btaf447_supplementary_data.docx]

**Table S1.** Optimal parameters of the networks in the training process.

| **Parameters** | **Optimal values** |
| --- | --- |
| **Learning rate** | **1e-4** |
| **Batch size** | **16** |
| **Dropout rate** | **0.1** |
| **Input channels** | **192** |
| **Output channels** | **96** |
| **Optimizer** | **Adam** |
| **Loss function** | **BCEFocalLoss** |

**Table S2.** Node representation of RNA secondary structures.

| '(' | '.' | ')' | '[' | ']' | '{' | '}' | '>' | '<' | 'A' or 'a' | 'B' or 'b' |
| --- | --- | --- | --- | --- | --- | --- | --- | --- | --- | --- |
| **0** | **1** | **2** | **3** | **4** | **5** | **6** | **7** | **8** | **9** | **10** |

**Table S3.** Performance of different features on T18.

| **Features** | **Precision** | **Recall** | **MCC** | **AUC** |
| --- | --- | --- | --- | --- |
| **TOR+ASA+SS** | 0.594 | 0.312 | 0.239 | 0.728 |
| **TOR+ASA** | 0.553 | 0.575 | 0.271 | 0.726 |
| **TOR+SS** | 0.635 | 0.288 | 0.250 | 0.684 |
| **ASA+SS** | 0.587 | 0.563 | 0.284 | 0.751 |
| **TOR** | 0.637 | 0.369 | 0.323 | 0.766 |
| **ASA** | 0.572 | 0.376 | 0.289 | 0.775 |
| **SS** | **0.705** | **0.706** | **0.636** | **0.902** |

**Table S4.** Performance of our method with different module compositions on T18.

| **Modules** | **Precision** | **Recall** | **MCC** | **AUC** |
| --- | --- | --- | --- | --- |
| **GAT** | 0.596 | 0.216 | 0.229 | 0.779 |
| **ERNIE-RNA** | 0.630 | 0.382 | 0.297 | 0.744 |
| **GAT+ERNIE-RNA** | **0.749** | **0.654** | **0.474** | **0.828** |
